# CT-severity score in COVID-19 patients: for whom is it applicable best?

**DOI:** 10.22088/cjim.13.0.228

**Published:** 2022

**Authors:** Alireza Almasi Nokiani, Razieh Shahnazari, Mohammad Amin Abbasi, Farshad Divsalar, Marzieh Bayazidi, Azadeh Sadatnaseri

**Affiliations:** 1 Department of Radiology, Firoozabadi Hospital, Iran University of Medical Sciences, Tehran, Iran; 2 Firoozabadi Clinical Research Development Unit (FCRDU), Iran University of Medical Sciences, Tehran, Iran; 3 Department of Cardiology, Sina Hospital, Tehran University of Medical Sciences, Tehran, Iran

**Keywords:** COVID-19, Computed Tomography, ROC Curve, Area Under Curve, CT-severity score

## Abstract

**Background:**

lung involvement in COVID-19 can be quantified by chest CT scan. We evaluated the triage and prognostication performance of seven proposed CT-severity score (CTSS) systems in two age groups of ≥65 and <65 years old.

**Methods:**

Confirmed COVID-19 patients by reverse transcriptase polymerase chain reaction (RT-PCR) admitted from February 20th, 2020 to July 22nd were included in a retrospective single center study. Clinical disease severity at presentation and at peak disease severity were recorded. CT images were scored according to seven different scoring systems (CTSS1-CTSS7). The cohort was divided into two age groups of ≥65 and <65 years old. Receiver operator characteristic (ROC) curves for each age group for diagnosis of severe/critical disease on admission (for triage) were plotted. Such curves were also plotted for predicting severe/critical disease at peak disease severity (for prognostication), and critical disease at peak severity (for prognostication). Areas under the curve (AUCs), best thresholds, and corresponding sensitivities (Sens.) and specificities (Spec.) were calculated.

**Results:**

96 patients were included with a mean age of 63.6±17.4 years. All CTSSs in 65-year-old or more group (N=55) showed excellent performance (AUC=0.80-0.83, Sens.+Spec.= 155-162%) in triage and excellent or outstanding performance (AUC=0.81-0.92, Sens.+Spec.= 153-177%) in prognostication. In the younger group (N=44), all CTSSs were unsatisfactory for triage (AUC=0.49-0.57) and predicting severe/critical disease (AUC=0.67-0.70), but were acceptable for predicting critical disease (AUC=0.70-0.73, Sens.+Spec.= 132-151%).

**Conclusion:**

CTSS is an excellent tool in triage and prognostication in patients with COVID-19 ≥65 years old, but is of limited value in younger patients.

## Introduction

**C**hest CT scan is a valuable tool in the initial evaluation and follow-up of COVID-19 patients and is strongly recommended (1). CT severity score (CTSS) has been used to quantify lung disease in COVID-19 with some triage and prognostication value. At least seven CTSS scoring systems have been proposed, all of which showing some success in triage and prognostication (2-11). They are presented in Table1. We have already reported a comparative study on the performance of these 7 different CTSS systems. The study had aimed to determine the value of CTSS in making decisions about the intensity of the treatment of respiratory failure (triage) and predicting the risk of development of severe/critical disease in the course of COVID-19 in correlation with selected clinical parameters (prognostication). 

We observed little difference in performance between the 7 scoring systems (ROC curve AUCs for triage = 0.67-0.7 and AUCs for prognostication = 0.76-0.79) and all of them showed good interrater reliability so that intraclass correlation coefficient (ICC) was 0.77-0.84 (11). To further analyze our cohort data, we decided to evaluate the performance of CTSSs in COVID-19 in different age groups in current study.

**Table 1:** seven proposed CT severity score systems

CTSSs	Segmentation	Severity Score for each segment	Maximum Score
CTSS1 (2,3)	Three zones in each lung are divided by carina and lower pulmonary vein	1-4 according to percentage of involvement (<25, 25-49, 50-74, ≥75)	24
CTSS2 (4)	The same zonal concept as CTSS1 with additional division of each zone into anterior and posterior regions divided by anteroposterior midpoint of diaphragm	1-4 according to percentage of involvement (<25, 25-49, 50-74, ≥75)	48
CTSS3 (5,6)	Five anatomic lobes of the lungs	1-4 according to percentage of involvement (<25, 25-49, 50-74, ≥75)	20
CTSS4 (7,8)	Five anatomic lobes of the lungs	1-5 according to percentage of involvement (<5, 5-24, 25-49, 50-74, ≥75)	25
CTSS5 (11)	Five anatomic lobes of the lungs with additional consideration of the lingula as a separate lobe	1-5 according to percentage of involvement (<5, 5-24, 25-49, 50-74, ≥75)	30
CTSS6 (9)	Five anatomic lobes of the lungs	1-4 according to the diameter of the largest lesion in each lobe (<1cm, 1-3cm, >3cm up to 50% of the lobe, >50% of a lobe	20
CTSS7 (10)	18 anatomic segments of the lung with an additional division of apicoposterior segment of the left upper lobe into apical and posterior divisions and anteromedial segment of the left lower lobe into anterior and medial segments	No involvement=0 <50% involvement=1 ≥50% involvement=2	40

## Methods

**Patients: **This study was approved by our institutional ethics committee (Approval ID: IR.IUMS.REC.1399.347). Our institutional review board waived the requirement to obtain written informed consent for this retrospective study, which evaluated de-identified data and involved no potential risk for patients. We enrolled patients with COVID-19 referred to Firoozabadi Hospital (Tehran, Iran) from February 20th, 2020 to July 22^nd^ who had at least one thoracic CT scan in our hospital. The diagnosis was based on positive results of reverse-transcriptase polymerase-chain-reaction (RT-PCR) assay of nasal and pharyngeal swab specimens at any time during hospitalization. Exclusion criteria were significant cardiopulmonary comorbidity, defined as cardiothoracic ratio >60% on CT topogram image (12) and diameter ratios of central branches of the pulmonary artery to corresponding bronchi >2 (13, 14); and pre-existing pulmonary disease involving more than 30% of the lungs, diagnosed subjectively by visual assessment of the same CT images by the radiologist (AAN). As RT-PCR was not routinely ordered for outpatients, our cohort included only hospitalized patients.

We collected clinical and laboratory data from the hospital information system (HIS), including disease severity at presentation, severity in the most severe disease period, outcome (death or discharge), place of hospital admission (ward or ICU), state of intubation, and any comorbidity.

The severity of the disease was decided by the information derived from patients’ records as is presented in Table 2 (15). For less complexity when the exact required data were not available, we regarded those who had undergone tracheal intubation or had died from the disease as critical. 

**Table 2:** clinical severity of COVID19

**Measured Indicator/Severity ** ^a^	**Mild**	**Moderate**	**Severe**	**Critical**
Respiratory Rate	≥24	≥30	-	-
SPO_2_	≥93	93>SPO_2_≥90	89>SPO_2_≥85	<85 ^b^
Respiratory Distress	None	None	Mild to moderate	Severe ^c^
Blood Pressure	-	-	-	<90/60

a: presence of any of the severity indicators of the more severe group places the patient in the more severe group

b: despite high-flow O_2_ administration

c: nasal flaring, air hunger, intercostal retraction, subcostal retraction

**Image acquisition:** Chest CT imaging was performed by a 16-detector-row CT scanner (Emotion; Siemens; Germany). All patients were examined in a supine position. CT images were acquired during a single inspiratory breath-hold. The scanning range was from the apex of the lung to the costophrenic angle. CT scan parameters: X-ray tube parameters - 110KVp, 45-60 effective mAs; rotation time - 0.6 seconds; collimation- 16x1.2; pitch - 1.5; section thickness – 5 mm; reconstruction interval – 5 mm with B70 sharp convolution kernel; additional reconstructions at slice thickness, and reconstruction interval of 1.5 mm with B70 and B31 convolution kernels were also made to generate lung and mediastinal windows, respectively. Lung window images were viewed at a width/level of 1200/-600 and mediastinal window images at 350/50 window settings.

**Image interpretation:** Two radiologists with 17 and 3 years of experience (AAN and RSh respectively) blinded to clinical data reviewed CT images of all the patients independently and scored each patient’s images according to each of the mentioned 7 scoring systems (CTSS1 to CTSS7). They viewed images on hospital PACS (Marco PACS Version 2.0.0.0) and resorted to multiplanar reconstruction (MPR) whenever needed. We took into account 10 of 14 imaging features defined in a previous study (15): ground-glass opacities (GGO), consolidation, mixed GGO and consolidation, centrilobular nodules, architectural distortion, tree-in-bud, bronchial wall thickening, reticulation, subpleural bands, and traction bronchiectasis. 

**Statistical Analysis:** All statistical analyses were done using SPSS 26.0 software (IBM, Armonk, NY), excluding comparison of ROC curves AUCs and selection of cut-off points which were conducted by MedCalc statistical software version 19.9.4.0. P<0.05 was considered statistically significant. Statistical analysis was performed by AAN. Quantitative data were expressed as mean ± standard deviation. 

ROC curve analysis was performed on the averages of reported CTSSs by the two raters for each CTSS to calculate the area under the curve (AUC) for diagnosis of severe/critical COVID-19 groups at the time of hospital admission (for triage). Then AUCs were classified unsatisfactory if AUC<0.7, acceptable if 0.7≤AUC<0.8, excellent if 0.8≤AUC<0.9, and outstanding if 0.9≤AUC (16). If AUC was acceptable or better, threshold, sensitivity (Sens.), and specificity (Spec.) for the CTSS and Sens.+Spec. were calculated. We chose the best thresholds according to the Youden index method which is choosing the threshold producing the largest Youden Index (Sens.+Spec. -1) (17). The same statistical procedure was used for the diagnosis of severe/critical disease and also for the diagnosis of critical disease at peak disease severity (for prognostication). 

We applied the same type of analysis for the patients aged 40 or more and again and again for each 5-year increment up to 75 or more, observing some progressive increase in AUCs with increasing age up to the 65 or more group; above which no further increase in AUCs was observed. Therefore, we divided our cohort using this age limit into a group including 55 patients aged 65 or more and a group of 41 patients aged 64 or less. Then we evaluated CTSS performance for each of the older and younger age groups separately.

## Results

**Figure 1:**
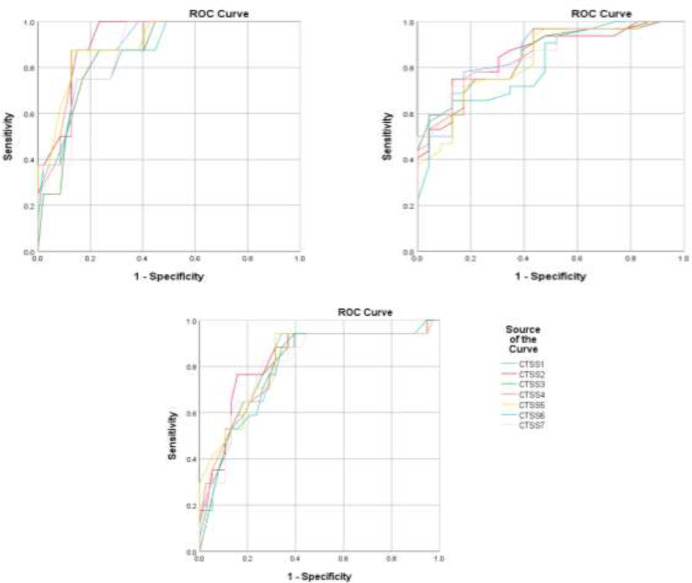
ROC curves plotted for different average CTSSs for diagnosing severe/critical disease at the time of hospital admission (top left), severe/critical disease at peak disease severity (top right), and critical disease at peak severity (bottom) in patients aged 65 or more

**Figure 2:**
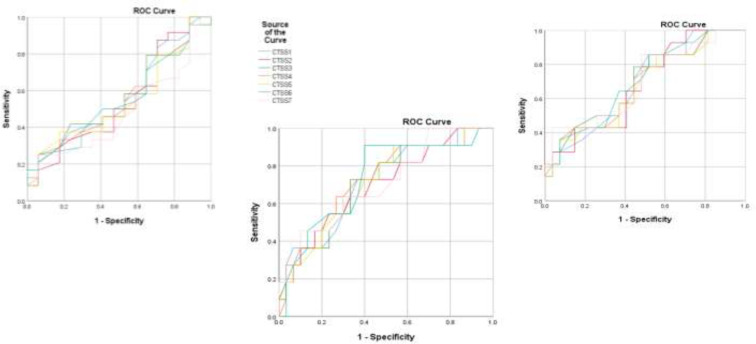
ROC curves plotted for different average CTSSs for diagnosing severe/critical disease at the time of hospital admission (top left), severe/critical disease at peak disease severity (top right), and critical disease at peak severity (bottom) in patients aged 64 or less

There were 145 cases confirmed by RT-PCR in the study period. Only 110 patients had at least one CT scan record in hospital picture archiving and communication system (PACS). 14 patients with cardiopulmonary comorbidity were excluded, consisting of 13 patients with heart failure and one patient with extensive centrilobular emphysema. The results of analysis of the whole cohort of 96 patients have been previously reported in detail (11). Only three scoring systems showed sufficient AUCs to be useful in triage (AUC=0.70, Sens.+Spec.= 131-132%). All CTSSs showed acceptable AUCs for prognostication in both diagnosing severe/critical disease (AUC=0.76-0.78, Sens.+Spec.= 140-146%) and diagnosing critical disease at peak disease severity (AUC=0.77-0.79, Sens.+Spec.= 141-146%) (11). In patients aged 65 or more, regarding AUCs of ROC curves for diagnosis of severe/critical disease at presentation, all the CTSSs were excellent (AUC=0.80-0.83, Sens.+Spec.= 155-162%); regarding AUCs for ROC curves for diagnosis of severe/critical disease at peak disease severity, CTSS1, CTSS2 and CTSS5 were outstanding (AUC=0.90-0.92, Sens.+Spec.= 173-177%) and the other CTSSs were excellent (AUC=0.86-0.89, Sens.+Spec.= 160-173%) and about AUCs for ROC curves for diagnosis of critical disease at peak disease severity, all the CTSSs were excellent (AUC=0.81-0.86, Sens.+Spec.= 153-162%). Corresponding ROC curves are shown in figure1 and the AUCs and confidence intervals, as well as related inference, chosen thresholds, sensitivity, and specificity for each CTSS, are presented in table3. In patients aged 64 or less, regarding AUCs of ROC curves for diagnosis of severe/critical disease at presentation, all the CTSSs were unsatisfactory for clinical implementation (AUC=0.49-0.57); regarding AUCs for ROC curves for diagnosis of severe/critical disease at peak disease severity all the CTSSs were also unsatisfactory (AUC=0.67-0.69), excluding CTSS6 with a borderline acceptable AUC value (AUC=0.70, Sens.+Spec.= 134%) and regarding AUCs for ROC curves for diagnosis of critical disease at peak disease severity, all the CTSSs were acceptable (AUC=0.70-0.73, Sens.+Spec.= 132-151%). Corresponding ROC curves are shown in figure 2 and the AUCs and confidence intervals, as well as related inference, chosen thresholds, sensitivity, and specificity for each CTSS, are presented in Table4. 

**Table 3:** AUC, confidence interval, related inference, best threshold, and related sensitivity and specificity and their sum for ROC curves for different CTSSs with respect to the diagnosis of severe/critical group at presentation (upper set) and at peak disease severity (middle set) and also for diagnosis of critical disease at peak severity (lower set) in patients aged 65 or more Sens.: Sensitivity, Spec.: Specificity

	**Average CTSS**	**AUC for ROC Curve**	**95% Confidence Interval**	**Inference about AUC**	**Best Threshold**	**Sens./ Spec. %**	**Sens.+ Spec. %**
diagnosis of severe/critical patients at presentation	CTSS1	0.81	0.68-0.94	excellent	11.0	66/94	160
	CTSS2	0.83	0.71-0.96	excellent	15.0	84/77	161
	CTSS3	0.80	0.67-0.94	excellent	10.5	61/94	155
	CTSS4	0.81	0.68-0.94	excellent	14.5	63/94	157
	CTSS5	0.82	0.69-0.95	excellent	15.5	68/94	162
	CTSS6	0.80	0.67-0.93	excellent	15.0	61/94	155
	CTSS7	0.81	0.68-0.94	excellent	18.5	74/82	156
diagnosis of severe/critical patients at peak disease severity	CTSS1	0.90	0.80-0.99	outstanding	7.5	85/88	173
	CTSS2	0.92	0.84-1.00	outstanding	15.0	77/100	177
	CTSS3	0.86	0.75-0.97	excellent	6.5	77/88	165
	CTSS4	0.89	0.79-0.99	excellent	10.0	85/88	173
	CTSS5	0.90	0.80-1.00	outstanding	11.5	87/88	175
	CTSS6	0.86	0.74-0.98	excellent	10.0	85/75	160
	CTSS7	0.87	0.77-0.98	excellent	18.5	66/100	166
diagnosis of critical patients at peak disease severity	CTSS1	0.86	0.70-0.88	excellent	9.5	78/83	161
	CTSS2	0.86	0.69-0.87	excellent	19.0	75/87	162
	CTSS3	0.85	0.69-0.87	excellent	12.5	59/96	155
	CTSS4	0.85	0.70-0.88	excellent	13.5	75/83	158
	CTSS5	0.83	0.68-0.86	excellent	15.5	72/83	155
	CTSS6	0.81	0.67-0.86	excellent	15.0	66/87	153
	CTSS7	0.86	0.70-0.88	excellent	22.0	72/87	159

**Table 4:** AUC, confidence interval, related inference, best threshold, and related sensitivity and specificity and their sum for ROC curves for different CTSSs with respect to the diagnosis of severe/critical group at presentation (upper set) and at peak disease severity (middle set) and also for diagnosis of critical disease at peak severity (lower set) in patients aged 64 or less Sens.= Sensitivity, Spec.=Specificity

	**Average CTSS**	**AUC for ROC Curve**	**95% Confidence Interval**	**Inference about AUC**	**Best Threshold**	**Sens./ Spec. %**	**Sens.+ Spec. %**
diagnosis of severe/critical patients at presentation	CTSS1	0.57	0.39-0.74	unsatisfactory	-	-	-
	CTSS2	0.54	0.36-0.72	unsatisfactory	-	-	-
	CTSS3	0.56	0.38-0.74	unsatisfactory	-	-	-
	CTSS4	0.55	0.37-0.73	unsatisfactory	-	-	-
	CTSS5	0.54	0.36-0.72	unsatisfactory	-	-	-
	CTSS6	0.55	0.37-0.73	unsatisfactory	-	-	-
	CTSS7	0.49	0.31-0.67	unsatisfactory	-	-	-
diagnosis of severe/critical patients at peak disease severity	CTSS1	0.69	0.52-0.86	unsatisfactory	-	-	-
	CTSS2	0.68	0.51-0.85	unsatisfactory	-	-	-
	CTSS3	0.69	0.52-0.86	unsatisfactory	-	-	-
	CTSS4	0.68	0.51-0.85	unsatisfactory	-	-	-
	CTSS5	0.67	0.49-0.84	unsatisfactory	-	-	-
	CTSS6	0.70	0.53-0.87	acceptable	16.0	48/86	134
	CTSS7	0.69	0.52-0.87	unsatisfactory	-	-	-
diagnosis of critical patients at peak disease severity	CTSS1	0.71	0.54-0.89	acceptable	10.5	73/63	136
	CTSS2	0.70	0.51-0.88	acceptable	24.5	55/77	132
	CTSS3	0.71	0.53-0.89	acceptable	9.5	73/67	140
	CTSS4	0.72	0.55-0.90	acceptable	15.0	64/73	137
	CTSS5	0.71	0.53-0.89	acceptable	13.0	91/47	138
	CTSS6	0.73	0.55-0.91	acceptable	12.5	91/60	151
	CTSS7	0.71	0.53-0.89	acceptable	28.5	46/90	136

## Discussion

Taking all age ranges into account in our reported study, only three scoring systems were acceptable for clinical use in triage of severe/critical disease patients and their performance is not very good as ROC curve AUCs were 0.67-070. CTSSs perform better in prognostication than triage with acceptable AUCs for all the CTSSs both in predicting severe/critical disease patients and predicting critically diseased patients at peak disease severity (ROC curve AUCs= 0.76-0.79) (11); Earlier studies reported more brilliant results. For example, Li and co-workers implemented CTSS3 and reported ROC curve AUC of 0.918 for diagnosis of severe/critical disease (6). Additionally, Yang and colleagues reported 0.892 for the same variable for CTSS7 (10). This discrepancy can be explained by much lower rates of severe/critical disease in the mentioned studies than our cohort; as their cohort included only about 10% severe/critical disease patients in the CTSS3 study (6) and less than 18% in the CTSS7 study (10), but in our study, the corresponding percentage was 57%. More recent reports show results compatible with our study as Hajiahmadi and colleagues reported ROC curve AUC 0.764 for CTSS1 for predicting severe/critical disease in a cohort including 24% severe/critical disease patients(18) while our calculated figure was 0.79 (11). In addition, Aminzadeh and co-workers used a CTSS method similar to our CTSS5 and reported ROC curve AUC of 0.65 for triage of severe/critical patients and 0.76 for predicting critical disease at peak disease severity (19) and our corresponding calculated values for CTSS5 were 0.69 and 0.77 respectively (11). To the best of our knowledge, our study is the first study assessing CTSS performance in triage and prognostication in COVID-19 in different age groups.

In the age range of 65-year-old or more, all CTSSs show excellent performance in the triage of severe/critical disease patients. CTSSs perform better in prognostication in this age group and three CTSSs namely CTSS1, CTSS2, and CTSS5 are outstanding in predicting severe/critical disease in the peak disease severity. All the other CTSSs are excellent in both predicting severe/critical and critical disease in peak disease severity.

In the age range of 64-year-old or less, CTSSs are not suitable for patients’ triage at all and are not essentially applicable for predicting severe/critical disease, excluding a borderline role for CTSS6. All CTSSs are acceptable for clinical use in predicting critical disease in this age group.

Two limitations should be considered, one is the absence of mildly diseased patients in our cohort which was because RT-PCR is not ordered routinely for mildly diseased patients who are not hospitalized. The other one was the absence of long-term follow-up after discharge to evaluate the relation of CTSSs to long-term sequelae of COVID-19. 

## Conclusion:

CT-severity score is an easy tool to quantify lung involvement. Considering all age groups, it has a limited value in the triage of severe/critical disease, but is acceptable as an indicator of prognosis. Accuracy of CTSS in triage and prognostication can be improved by dividing patients in two ≥65 and <65 years old age groups. In patients 65-year-old or older, CT-severity score shows excellent performance in both triage and prognostication. In patients aged 64 or less, CT-severity score has almost no value in triage and a limited value in prognostication. 
